# Plant endophytic fungi exhibit diverse biotransformation pathways of mogrosides and show great potential application in siamenoside I production

**DOI:** 10.1186/s40643-024-00754-8

**Published:** 2024-04-23

**Authors:** Wenxi Lin, Qiang Jiang, Yamin Dong, Yiwen Xiao, Ya Wang, Boliang Gao, Du Zhu

**Affiliations:** 1 Key Lab of Bioprocess Engineering of Jiangxi Province, College of Life Sciences, Jiangxi Science and Technology Normal University, Nanchang, 330013 China; 2 Key Laboratory of Protection and Utilization of Subtropic Plant Resources of Jiangxi Province, Jiangxi Normal University, Nanchang, 330022 China

**Keywords:** Bioconversion, Mogrosides, Fermentation, Biocatalysis, Fungal endophytes

## Abstract

Fungal endophytes, as an untapped resource of glycoside hydrolase biocatalysts, need to be further developed. Mogroside V, the primary active compound in *Siraitia grosvenorii* fruit, can be converted into other various bioactive mogrosides by selective hydrolysis of glucose residues at C3 and C24 positions. In present study, 20 fungal strains were randomly selected from our endophytic fungal strain library to assess their capability for mogroside V transformation. The results revealed that relatively high rate (30%) endophytic fungal strains exhibited transformative potential. Further analysis indicated that endophytic fungi could produce abundant mogrosides, and the pathways for biotransforming mogroside V showed diverse. Among the given fungal endophytes, *Aspergillus* sp. S125 almost completely converted mogroside V into the end-products mogroside II A and aglycone within just 2 days of fermentation; *Muyocopron* sp. A5 produced rich intermediate products, including siamenoside I, and the end-product mogroside II E. Subsequently, we optimized the fermentation conditions for *Aspergillus* sp. S125 and *Muyocopron* sp. A5 to evaluate the feasibility of large-scale mogroside V conversion. After optimization, *Aspergillus* sp. S125 converted 10 g/L of mogroside V into 4.5 g/L of mogroside II A and 3.6 g/L of aglycone after 3 days of fermentation, whereas *Muyocopron* sp. A5 selectively produced 4.88 g/L of siamenoside I from 7.5 g/L of mogroside V after 36 h of fermentation. This study not only identifies highly effective biocatalytic candidates for mogrosides transformation, but also strongly suggests the potential of plant endophytic fungi as valuable resources for the biocatalysis of natural compounds.

**Supplementary Information:**

The online version contains supplementary material available at 10.1186/s40643-024-00754-8.

## Introduction

Endophytic fungi are an ecological group of fungi that inhabit the tissues and organs of healthy plants, without causing any external symptoms of the host plant (Staniek et al. 2008). In last decades, endophytic fungi have been attracted more and more attention due to its excellent capacity for secreting a diverse array of bioactive compounds and enzymes (Wang et al. 2023). Especially, these enzymes secreted, which are used for plant tissue invasion, colonization, and nutrient acquisition by endophytic fungi, make them a valuable source of biocatalysts for the biotransformation of natural products. (Higginbotham et al. 2013; Suryanarayanan et al. 2012; Gao et al. 2021; Xiao et al. 2022). Recently, some studies have found that plant fungal endophytes exhibit excellent properties in bioconversion of natural saponin, such as glycyrrhizic acid (Gao et al. 2021; Xiao et al. 2022), ginsenosides (Eom et al. 2018), with high activity and substrate specificity. Indeed, fungal endophytes have been suggested as high-efficient biocatalyst sources, revealing great potential application in medicine, food security, and social sustainability (Schulz et al. 2002; Suryanarayanan et al. 2012; Choudhary et al. 2021). However, comprehensive information on the chemical conversion capabilities of most endophytic fungal genera for biotechnological applications remains scarce thus far.

**Fig. 1:**
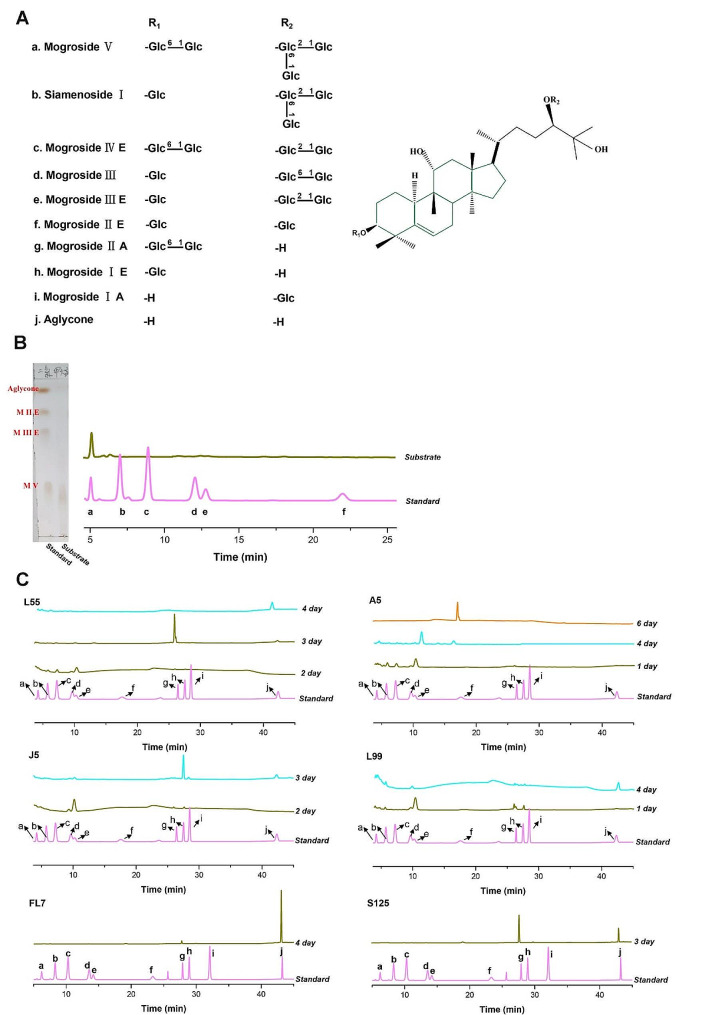
HPLC analysis of products produced by bioconversion mogroside V of 6 selected fungal endophytes. **A** the chemical structure of different mogrosides; **B** the substrate containing 0.5% (w/v) Luo Han Guo extract (LHG extract); **C** Strains L55, A5, J5, L99, FL7, and S125 were cultured and fermented in a medium containing 0.5% (w/v) Luo Han Guo extract (LHG extract). The fermentation supernatant was collected per day, and the mogrosides in the fermentation broth were analyzed using HPLC method. The standards as following: (a) mogroside V; (b) siamenoside I; (c) mogroside III E; (d) mogroside III; (e) mogroside III E; (f) mogroside I E; (g) mogroside I A; (h) mogroside I E; (i) mogroside I A; (j) aglycone

*Siraitia grosvenorii*, a perennial vine belonging to *Cucurbitaceous*, is an indigenous plant of China and primarily found in the provinces of Guangxi, Guangdong and Jiangxi (Wang et al. 2019). For centuries, its fruits, called as luo han guo (LHG), have been utilized in traditional Chinese medicine for treating constipation, lung congestion, dry cough, etc. (Takasaki et al. 2003). LHG contains a diverse array of bioactive compounds, such as flavonoids, alkaloids, polysaccharides, vitamins, aliphatic acids and triterpene saponins (Shivani Thakur et al. 2021). Of particular significance, mogrosides, a group of cucurbitane-type triterpene saponins (Fig. 1A), are the principal pharmacological components of LHG, and also are widely used as sugar substitute in the world due to their high sweetness and low calorie (FDA 2010). In fact, the relative sweetness of mogroside V, mogroside IV, siamenoside I and mogroside II were assayed to be 378, 300, 465 and 195 times sweetness than sucrose in water, respectively. Among these mogrosides, siamenoside I shows the highest sweetness and most acceptable taste quality (Muñoz-Labrador et al. 2021). Currently, numerous studies have revealed various biological functions of mogrosides, including anti-inflammatory (Qi et al. 2008; Liu et al. 2021), antioxidation (Chen et al. 2007), anti-tumor (Liu et al. 2015), liver protection (Shi et al. 2014) and modulation of glycolipid metabolism (Liu et al. 2018). Overall, existing research strongly suggests that mogrosides are promising candidates for the development of novel drugs and high-sweetness natural sweeteners (Wu et al. 2022).

Mogrosides represent a diverse array of derivatives with intricate stereoconfigurations, consisting of both aglycone (cucurbitane-type triterpene) and 1 to 5 glucose moieties (Fig. 1A) (Chiu et al. 2013). These derivatives, extracted from LHG, primarily include mogroside V, mogroside IV, siamenoside I, mogroside III, mogroside II, and mogroside I (Fig. 1A) (Gong et al. 2019). Among these, mogroside V constitutes the principal component (∼60%, w/w) of total saponins in LHG (Pawar et al. 2013), the contents of other mogrosides, however, are considerably low. Therefore, other mogrosides are mainly produced by the hydrolysis of mogroside V. Unfortunately, due to the intricate sugar moieties in mogroside V, producing the desired products by the traditional chemical hydrolysis methods, such as acid hydrolysis, proves to be challenging (Bin et al. 2020). In contrast, biotransformation approach offers significant advantages in terms of spatial stereoselectivity, sustainability, and environmental friendliness, and has been considered as more potential strategy for mogroside derivative production (Li et al. 2022). Given the excellent sweetness intensity and good taste of siamenoside I, its bioconversion has recently garnered attention (Xu et al. 2021; Duan et al. 2023). However, the screening of biocatalytic candidates capable of converting mogroside V into siamenoside I remains limited, and those that do exist are often lacking in selectivity and efficiency for this transformation. In addition, there are also few reports on the biotransformation of other rare mogrosides, including mogroside II, mogroside I, and mogroside III, leading to hinder further investigation of these mogrosides. Indeed, existing studies predominantly focus on catalytic enzymes, while ignore the screening of strains, which is the source of efficient and specific enzyme. Furthermore, in some aspects, biotransformation using whole-cell catalysis maybe exhibit advantages over enzyme catalysis because of its lower cost and higher stability (Xu et al. 2022).

In our previous study, a total of 229 strains of endophytic fungi belonging to 19 genera were isolated from Dongxiang wild rice (*Oryza rufipogon* Griff.) (Wang et al. 2015). Further investigation revealed that these endophytic fungi could secreted abundant glycoside hydrolase, suggesting great potential application in biotransformation of the natural products, especially glycosides (Gao et al. 2021, 2023). In the present study, we randomly selected 20 strains from our fungal endophyte library for screening the ability of mogrosides transformation. Our results revealed that a high proportion of these strains show the capacity for converting mogroside V into other glycosides. Furthermore, two strains, A5 and S125, exhibiting high conversion efficiency and β-glucosidase activity, were selected to produce various mogroside derivatives. This study not only provides diverse biocatalytic candidates for mogroside bioconversion which significantly facilitate the development of mogroside industry, but also demonstrates that endophytic fungi are a great potential biocatalytic resource for natural products.

## Materials and methods

### Medium and chemicals

The primary culture medium consisted of potato dextrose agar (PDA), comprising 200 g/L of potato, 20 g/L of glucose, and 20 g/L of agar powder. For strain screening, the culture medium comprised the following components: 5.0 g of LHG extract, 2.0 g of KH_2_PO_4,_ 5.0 g of NH_4_NO_3,_ 0.5 g of NaCl, 0.05 g of yeast extract, 0.4 g of MgSO_4_·7H_2_O, 0.04 g of ZnSO_4_·7H_2_O, and 1 L of sterile water, adjusted pH 6.0. The LHG extract (containing 50% of mogroside V) was purchased from Luoyang Tianluo Biological Co., LTD (China). The standard products of mogroside V, IV E, III A, III E, II A, II E, I A, I E, siamenoside I and aglycone (purity, 98%) were obtained from Chengdou Munster Biotechnology Co., LTD (China). All other chemicals used in this study were of analytical grade standards.

### Strain screening

A total of 229 strains of endophytic fungi involving 19 genera were previously isolated from Dongxiang wild rice (Wang et al. 2015). Subsequently, 20 strains were randomly selected from the endophytic fungal library for mogroside V transformation. Verification of mogaloside V hydrolyzing ability was carried out using extracellular enzymes produced through the submerged fermentation of these 20 fungi. Fungal activation performed either on PDA or PDB, and the fungal mycelia of the selected 20 strains were inoculated at a concentration of 2% (v/w) into an inorganic medium containing 5% (w/v) LHG extract as the sole carbon source. Aerobic fermentation was performed at 28℃ and 160 rpm in a shaker over a 7-day period. Broth samples were collected every 24 h during the fermentation, and the supernatant was acquired by centrifugation at 4℃, 7000 rpm. Product analysis of the supernatant was performed using a thin-layer chromatographic plate (TLC) with a developing agent consisting of dichloromethane: methanol in a 5:3 (v/v) ratio, and a color developer composed of a 10% ethanol sulfate solution. High-performance liquid chromatography (HPLC, Agilent, USA) was employed to further identify the hydrolysis products of mogroside V produced by these strains. Strains exhibiting conversion ability were then selected for further investigation.

### Morphological and phylogenetic analysis of strains

After screening test, a total of 6 endophytic fungi, namely strains S125, FL7, L55, A5, L99, and J5, capable of utilizing LHG extracts for growth were identified for microorganism identification. The colony morphology of endophytic fungi was observed on PDA medium at 28℃, while the morphology of spores, sporangia, and spore chains were examined using VEGA3 scanning electron microscopy (Tescan, USA). Genomic DNA extraction from the given fungal strains utilized the cetyltrimethylammonium bromide (CTAB) method (Van burik et al., 1998). Primers ITS1 (5’- TCCGTAGGTGAACCTGCGG-3’) and ITS4 (5’-TCCTCCGCTTATTGATATGC-3’) were utilized for amplifying internal transcriptional spacers (ITS). PCR was employed to obtain the amplified DNA fragments, and sequencing was carried out by Qingke Biological Company (China). Phylogenetic analysis of the ITS-rDNA sequences of these 6 endophytic fungi was carried out. The ITS sequences of endophytic fungi were compared online with NCBI (http://www.ncbi.nlm.nih.gov/BLAST) using the BLAST (Basic Local Alignment Search Tool) to ascertain the genetic relationships and classification status of the endophytic fungi. Sequence data for relevant species were downloaded based on GenBank results. MEGA software (6.0.6) was utilized for comparing multiple sequences and constructing a phylogenetic tree based on evolutionary distance data. The tree figure was manually edited using iTOL version 4.

### Optimization of fermentation conditions for mogrosides bioconversion

Two endophytic strains, S125 and A5, were selected for further investigation. The optimization of substrate concentration, nitrogen source type, and nitrogen source concentration for β-glucosidase production by strains S125 and A5 was carried out using a one-by-one method. The fundamental fermentation conditions were as follows: 5 g/L of LHG extract (w/v), 5 g/L of NH_4_NO_3_ (w/v), 2.0 of g/L KH_2_PO_4,_ 0.5 g/L of NaCl, 0.4 g/L of MgSO_4_·7H_2_O, 0.04 g/L of ZnSO_4_·7H_2_O, and a natural pH. The seed broth was inoculated into the basic fermentation medium at a 2% inoculation rate, with a volume of 50 mL per 250 mL flask, and fermented at 28℃ and 160 rpm for 7 days. Subsequently, enzyme activity was determined using the method described by Javed et al. (2018). The effect of carbon and nitrogen source concentrations on β-glucosidase activity was investigated through a single-factor test in the basic medium with LHG extract as the sole carbon source, respectively. Under the basic fermentation conditions, the concentration gradient ranged from 1 g/L to 30 g/L to explore the effects of different substrate concentrations on β-glucosidase activity. To examine the influence of various nitrogen sources on β-glucosidase activity, variations such as 0.5% (w/v) of NaNO_3_, NH_4_NO_3_, NH_4_Cl, beef extract, peptone, urea, and yeast powder were employed to replace the nitrogen source in the basic fermentation medium, respectively. Subsequently, the optimal nitrogen source was selected, and its concentration was varied from 1 g/L to 11 g/L to investigate the effects of different nitrogen source dosages on β-glucosidase activity. Finally, the production of mogrosides by the fermentation of strains S125 and A5 was carried out under optimal conditions, respectively.

### Mogroside analysis by HPLC

The fermentable broth obtained from the given endophytic fungi was collected for further analysis. Initially, the fermentative supernatant was separated via filtration, followed by the addition of an equal volume of n-butanol for extraction. After thorough oscillation and mixing, the organic residue was evaporated and dried using a rotary evaporator, then dissolved in methanol and filtered through a 0.22 μm membrane for HPLC analysis. Standard products of mogroside V, IV E, III E, III, II A, II E, I A, I E, siamenoside I, and aglycone were dissolved in 10 mL of chromatography-grade methanol to create a 1 mg/mL standard solution. The contrast solution of mogrosides with different concentration gradients was prepared from the mother liquor and analyzed by HPLC to investigate its linear relationship and range. The standard curve was constructed with the mass concentration (X, µg/mL) of the control solution as the X coordinate and the peak area (Y, AU*s) as the Y coordinate, the standard curve was drawn and the regression. Chromatographic conditions included an SB-C18 column (150 mm×4.6 mm, 5 μm) with a wavelength of 203 nm, a flow rate of 0.8 mL/min, a sample volume of 10 µL, and acetonitrile: water as the mobile phase in a gradient elution ranging from 2.8:7.2 to 6.5:3.5. By comparing the retention time of each standard with the samples, the presences of different types of mogrosides in the fermentation broth were determined. The content of mogrosides in the sample was calculated by regression curve of standard mogrosides.

### Statistical analysis

All experiments were conducted in triplicate with the average value being reported on the dry basis. The differences between variables were tested for significance using ANOVA and Duncan’s multiple range test. Differences between means were considered significantly different at *P* < 0.05.

## Results and discussions

### Screening fungal endophytes for bioconversion of mogrosides

Recently, some microorganism, mainly yeast, have been employed to transform mogrosides due to their glucosidase activity, showing the ability for converting mogroside V into a mixture of mogroside IV, siamenoside I, and mogroside III E (Chiu et al. 2013; Wang et al. 2019). According to our previous studies, fungal endophytes secreted rich glycosidases and exhibited the ability to transform terpenoid saponins, such as glycyrrhizic acid (Xiao et al. 2022). Therefore, we supposed that the fungal endophytes maybe have the capacity to transform mogrosides. To investigate this hypothesis, 20 strains from our endophytic fungi library were randomly selected to transform the LHG extracts (containing 50% mogroside V). After screening test, a total of 6 endophytic fungi capable of utilizing LHG extracts for growth were found, suggesting their potential for mogrosides conversion. Subsequently, fermentation of the 6 strains using LHG extracts was carried out, respectively, and fermentable supernatant were sampled daily for further analysis using the TLC method. As shown in S Fig. 1, the 6 given strains exhibited diverse mogroside profiles, revealing diverse and excellent mogroside bioconversion abilities. Notably, strain S125 exhibited excellent ability for selective efficient bioconversion of mogrisde V into two products, meaning great potential for specific production of rare mogrosides. Meanwhile, diverse intermediate products were produced during fermentation of strain FL7, L55, A5, L99, and J5, with one type of mogroside derivative predominantly obtained in the middle or late fermentation stages. Previously, various microorganisms, including yeast and lactic acid bacteria, were tested for mogrosides conversion, but only a few yeasts showed this ability (Wang et al. 2018; Yang et al. 2007). Thus, the screening of novel biocatalytic candidates for efficient mogroside conversion is necessary. When comparing our results to those previous studies, it must be pointed out that a relatively high ratio (approximately 30%) of fungal endophytes with mogroside conversion capabilities were screened, and these given fungal endophytes also exhibited excellent properties in producing diverse mogroside derivatives. Indeed, our results strongly suggest plant endophytic fungi are promising biocatalytic candidates for mogroside conversion.

### Diverse pathways for biotransformation of mogrosides by fungal endophytes

To further identify the intermediate or end products, HPLC analysis was employed. Our results showed that most of the tested fungal endophytes, except for strain A5, completely converted mogroside V to aglycone as the end-product (Fig. 1B and C). Currently, reported biocatalytic strains typically transform mogroside V to mogroside III E as the end-product (Table 1). Similarly, mogroside III E remained the main component of products generated during the middle fermentation period of fungal endophytes, such as strain L55, A5, J5 and L99. Meanwhile, we also found the production of siamenoside I during the early fermentation period of strain A5. During the middle fermentation period (2∼4 days) of strain L55 and FL7, relatively high content of mogroside I A was produced. In addition, strain J5 exhibited a quite specific property, producing a rare mogroside namely mogroside I A, during the middle fermentation period. To our knowledge, there are no reports of strains or enzymes capable of converting mogroside V into mogroside I E. Surprisingly, strain S125 exhibited high conversion efficiency, utilizing almost all substrates to produce end-products (mogroside I A, and aglycone) after only one day of fermentation, demonstrating great potential application for the production of rare mogrosides.

**Table 1:** Bioconversion of mogroside by biocatalytic candidates in 7 days

Strains	Substrate	Intermediate products	End-products	Reference
*Ganoderma lucidum*	LHG extracts	M III E, M II A	M III E, M II A	Chiu et al. 2019
*Kluyveromyces marxianus*	LHG extracts	S I, M III E	S I, M III E	Wang et al. 2018
*Saccharomyces pastorianus*	LHG extracts	M III E	M III E	Wang et al. 2018
*Candida kefyr*	LHG extracts	S I, M III E	S I, M III E	Wang et al. 2018
*Candida utilis*	LHG extracts	S I, M III E	S I, M III E	Wang et al. 2018
*Yarrowia lipolytica*	LHG extracts	S I, M III E	S I, M III E	Wang et al. 2018
*Debaryomyces hansenii*	LHG extracts	S I, M III E	S I, M III E	Wang et al. 2018
*Dekkera bruxellensis*	LHG extracts	S I, M III E	S I, M III E	Wang et al. 2018
*Muyocopron* sp. A5	LHG extracts	S I, M III E, M III E, M II A	M II A	This study
*Alternaria* sp. FL7	LHG extracts	M II A, aglycone	Aglycone	This study
*Sarocladium oryzae* L99	LHG extracts	M III E, M II A, M I E	Aglycone	This study
*P. meleagrinum* J5	LHG extracts	M III E, M III, M I E	Aglycone	This study
*Aspergillus* sp. L55	LHG extracts	M IV E, M III E, M II A	Aglycone	This study
*Aspergillus* sp. S125	LHG extracts	M II A, aglycone	M II A, aglycone	This study

*Notes* mogroside and siamenoside were abbreviated as M and S, respectively

**Fig. 2:**
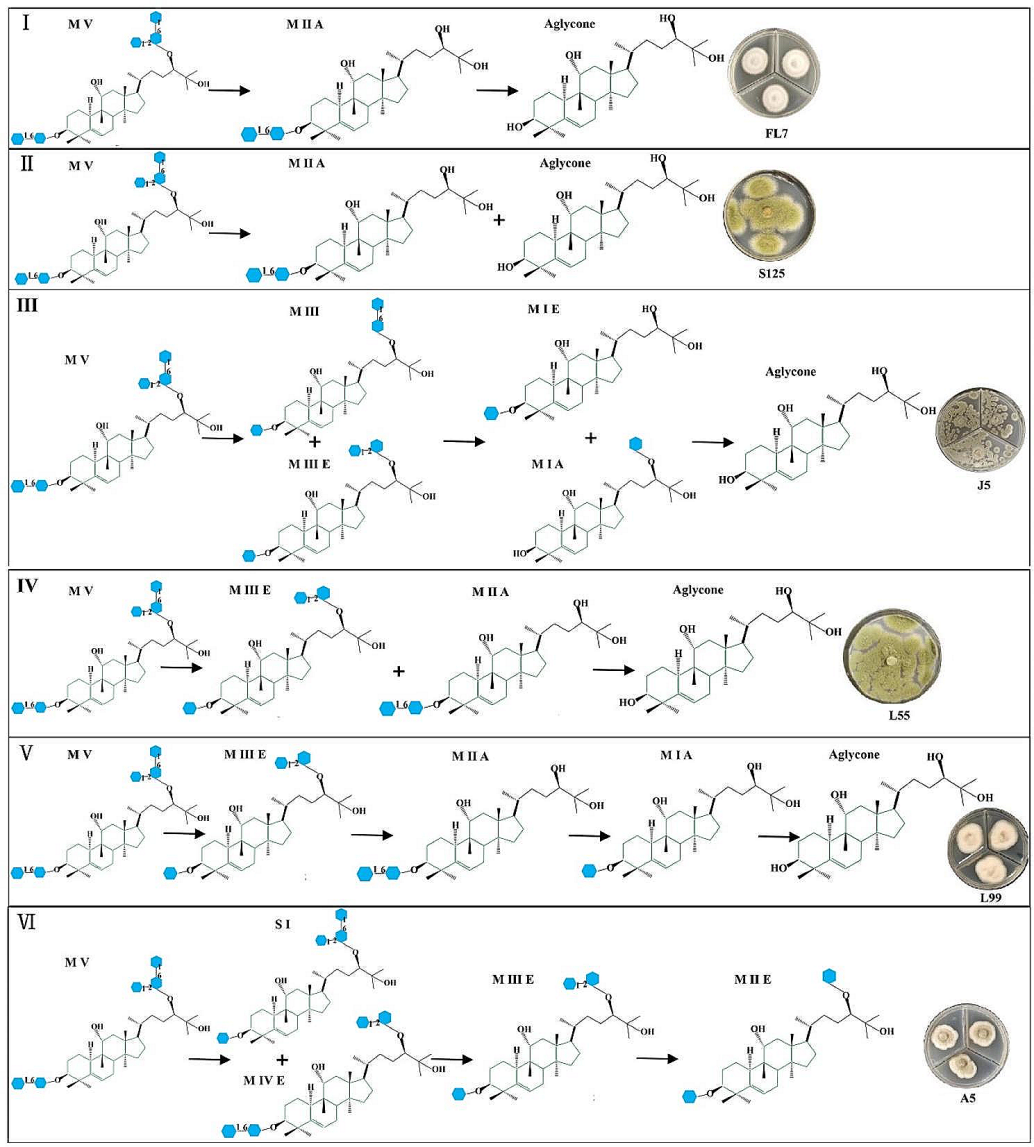
The bioconversion pathways of mogroside V during the conversing processes by endophytic fungi L55, A5, J5, L99, FL7 and S125. Mogroside and siamenoside were abbreviated as M and S, respectively

Evidences indicate that various bioactive compounds can be obtained by selectively removing glucose moieties from mogroside V at the C3 or C24 positions. These compounds exhibit diverse biological activities, such as tumor inhibition and high sweetness as a sweetener (Liu et al. 2015; Wu et al. 2022). However, mogroside V contains relatively complex glucose moieties, resulting in challenges of the targeted preparation of certain LHG glycosides. As a result, bioconversion strategies with high selectivity are preferred. Few studies have investigated the biotransformation of mogrosides, with most focusing on yeast-mediated bioconversion of mogroside V. For example, Wang et al. (2018) screened microorganisms capable of converting mogroside V, identifying 8 different yeast strains with transformative abilities. Their results revealed that the main products produced by yeasts were siamenoside I and mogroside III E. It seems to be suggested a consistent pathway for yeast-mediated mogroside V conversion, namely M V → S I + M III E (herein, mogroside and siamenoside were abbreviated as M and S, respectively). Comparing to yeast, utilizing endophytic fungi as catalysts for the biotransformation of mogroside V results in a more diverse conversion pathway (Fig. 2), including (i) M V → M I A → M A (FL7); (ii) M V→ M I A + M A (S125); (iii) M V → M III E + M III → M I A + M I E → M A (J5); (iv) M V → M III E + M I A → M A (L55); (v) M V → S I + M III E → M I A + M I E → M A (L99); (vi) M V → S I + M III E → M III E → M I E (A5), and a richer array of products (Figs. 1 and 2). Not only can similar products be generated, but also rare products, such as mogroside I A and aglycone, which are unattainable by yeast conversion, can also be obtained by plant endophytes. From the above mentioned results, endophytic fungi can be considered as superior biocatalysts for the bioconversion of mogrosides.

### Phylogenetic analysis of endophytic fungi with capacity of mogrosides biotransformation

**Fig. 3:**
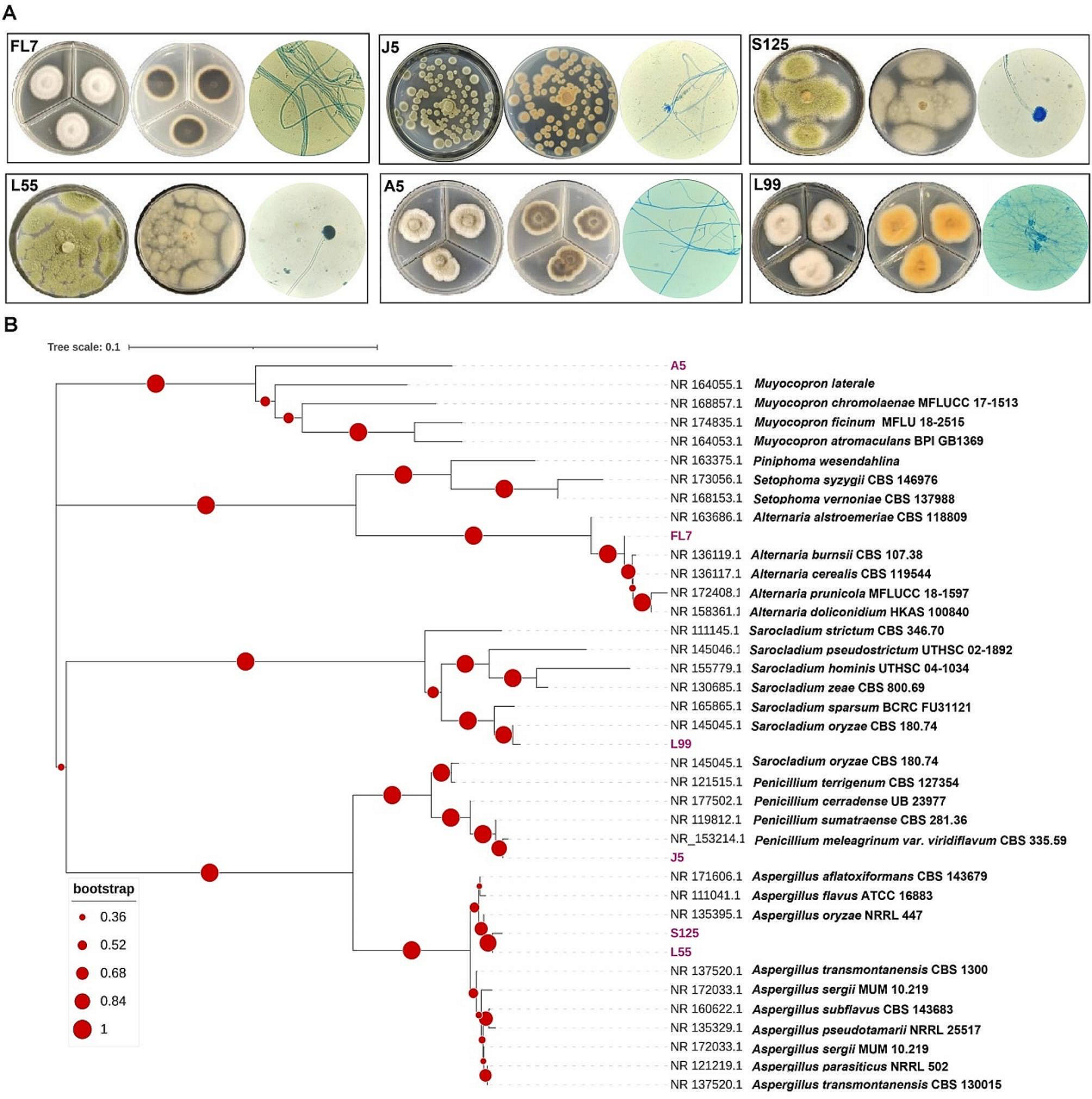
**A** Morphological characteristics of endophytic fungi L55, A5, J5, L99, FL7 and S125, and **B** Maximum-likelihood (ML) trees based on ITS sequences using MEGA software (version 6.0.6) with the Kimura 2-parameter model for calculations of evolutionary distances. The bootstrap values (1000 replicate runs) greater than 50% are listed

Microorganism identification is considered necessary for further investigations of transformation characteristics, catalytic enzymes, and strain screening. Therefore, the 6 strains exhibiting mogroside bioconversion capabilities were further identified. Based on morphological characteristics (Fig. 3A), strains S125 and L55 grow rapidly with abundant aerial hyphae covering the entire agar plate, and produce numerous conidiophores, suggesting they belong to the genus *Aspergillus*. In contrast, strain J5 exhibits distinct septa in both its hyphae and conidiophores, indicating potential classification within the genus *Penicillium*. Strains FL7 and A5 display filamentous mycelium without noticeable spores. To further identify these filamentous fungi, molecular taxonomy, a rapid and reliable method, was employed. The molecular phylogenetic tree was constructed based on ITS regions (Fig. 3B). All members of *Muyocopron* species were grouped in a single cluster, and the Fungus A5 placed on a single clade, revealing a potential novel species of the genus *Muyocopron.* Although fungus FL7 belongs to the genus *Alternaria* which showed the highest similarity with *Alternaria alstroemeriae* CBS 118,809 ITS region (Genebank accession no. NR_163686.1), fungus FL7 gathered a single clade, demonstrating a potential novel species of *Alternaria.* Additionally, fungus L99 and J5 were designated as *Sarocladium oryzae* L99 and *P. meleagrinum* J5, respectively, due to high sequences identity (≥ 99%) of sequenced ITS with those from *Sarocladium oryzae* CBS 180.74 (Genebank accession no. NR_145045.1), and *P. meleagrinum* var. viridiflavum CBS 335.59 (Genebank accession no. NR_153214.1), respectively. Importantly, two strains, namely fungal strains L55 and S125 were grouped in a cluster with numbers of genus *Aspergillus*, which revealed two strains all belonging to *Aspergillus* species. Our results showed that three strains capable of transforming mogrosides belong to the genera of *Penicillium* and *Aspergillus* fungi, with a proportion reaching 50%. In addition to the hydrolytic activity towards mogrosides described in this paper, previous studies have revealed that *Penicillium* or *Aspergillus* fungi exhibited transformative capabilities for various natural glycoside compounds, such as glycyrrhizic acid and saponins (Zou et al. 2013; Liu et al. 2013; Lee et al. 2021). This may be attributed to the rich glycoside hydrolase harboring in *Penicillium* and *Aspergillus* fungi, indicating their potential for transforming a variety of glycoside compounds.

### Production of rare mogrosides from the LHG extracts

**Fig. 4:**
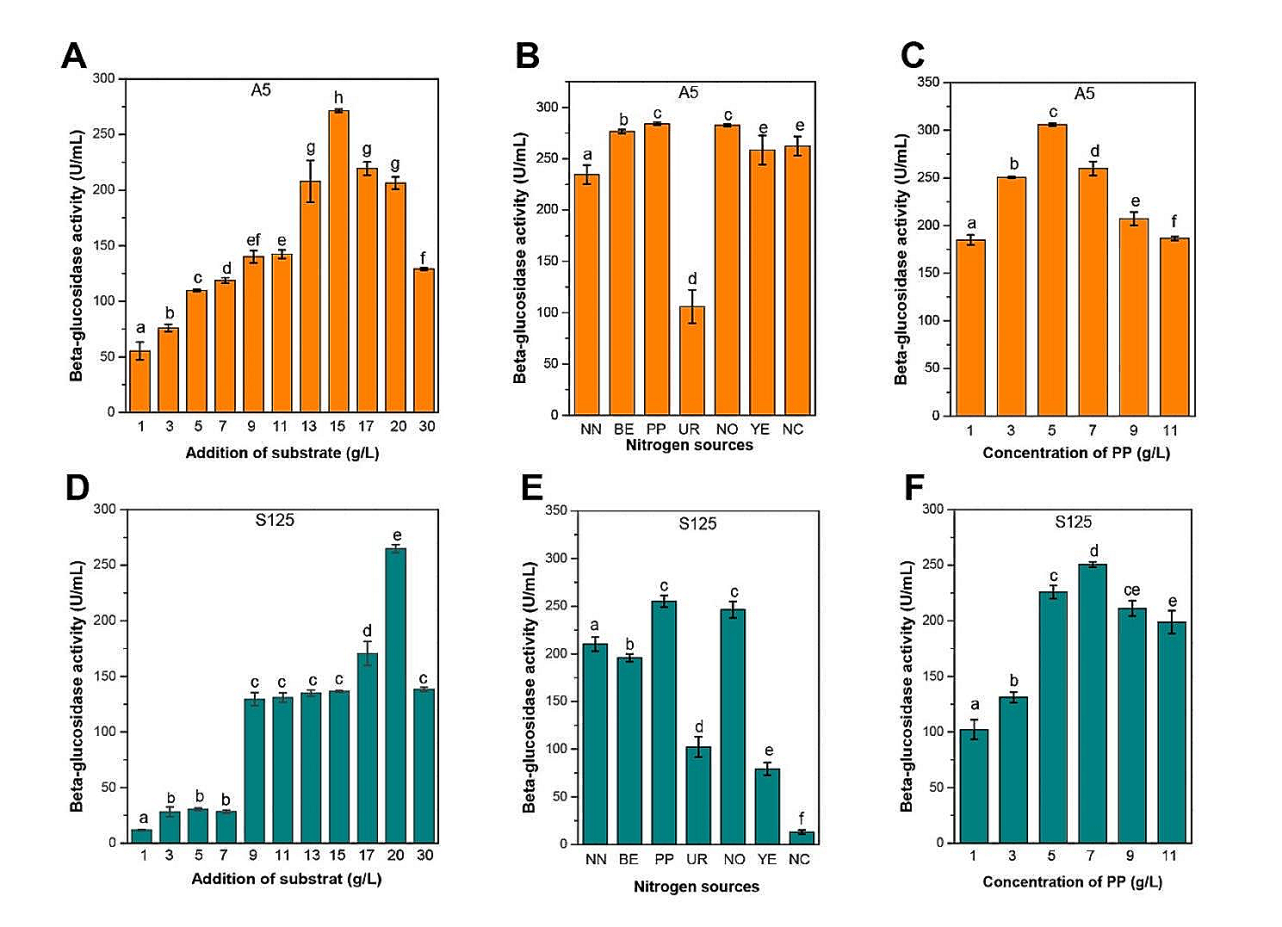
Effect of **A, D** LHG extract dosages, **B, E** different nitrogen sources and **C, F** concentration of peptone on β-glucosidase activities of *Muyocopron* sp. A5 and *Aspergillus* sp. S125, respectively. NN, NaNO_3_; BE, beef extract; PP, peptone; UR, urea; NO, NH_4_NO_3_; YE, yeast powder; NC, NH_4_Cl

According to our above results, diverse derivatives of mogroside were produced from the bioconversion of mogroside V by plant endophytic fungi. Compared to other fungi, *Aspergillus* sp. S125 could fast transform mogroside V into two end-products (mogroside I A and mogroside saponin, Fig. 1B**)**. Among the various mogrosides, siamenoside I not only exhibits the highest sweetness but also the most optimal taste, making it widely recognized as one of the most promising natural sweeteners. Therefore, it is of great significance to find and develop biocatalysts that can effectively convert mogroside V into siamenoside I. Fortunately, during the early stage of fermentation, *Muyocopron* sp. A5 efficiently converted mogroside V into siamenoside I (Fig. 1B). To further evaluate the feasibility of mogroside production, two strains, namely *Muyocopron* sp. A5 and *Aspergillus* sp. S125, were selected to produce corresponding mogrosides. Previous studies have shown high levels of β-glucosidase activities, meaning excellent bioconversion ability for mogrosides (Chen et al. 2022), as this glycosidase is usually responsible for hydrolyzing the glycosidic bonds of mogrosides. Thus, β-glucosidase activities were selected as a parameter to optimize cultural conditions. Different amounts of substrates (ranging from 1 to 30 g/L of LHG extract), and various nitrogen sources were employed to culture *Muyocopron* sp. A5 and *Aspergillus* sp. S125, respectively. Our results demonstrated a significant effect of substrates and nitrogen sources on β-glucosidase activity produced by the two selected strains (Fig. 4). After 7 days of fermentation, the highest β-glucosidase activity (190.15 U/mL) was generated by *Muyocopron* sp. A5 cultivated in medium containing 15 g/L of LHG extract (Fig. 4A). In addition, various nitrogen sources, including NaNO_3_, beef extract, peptone, urea, NH_4_NO_3_, yeast powder, and NH_4_Cl, were employed to culture *Muyocopron* sp. A5, and the highest β-glucosidase activity (284.17 U/mL) was assayed at peptone (Fig. 4B). Subsequently, different dosages of peptone (1∼11 g/L) were further used to culture *Muyocopron* sp. A5, and our results showed that 5 g/L of peptone contributed to the highest β-glucosidase activity (306.03 U/mL) (Fig. 4C). Meanwhile, as shown in Fig. 4D∼F, 20 g/L of LHG extract and 7 g/L of peptone caused the highest β-glucosidase activity for *Aspergillus* sp. S125, respectively.

**Fig. 5:**
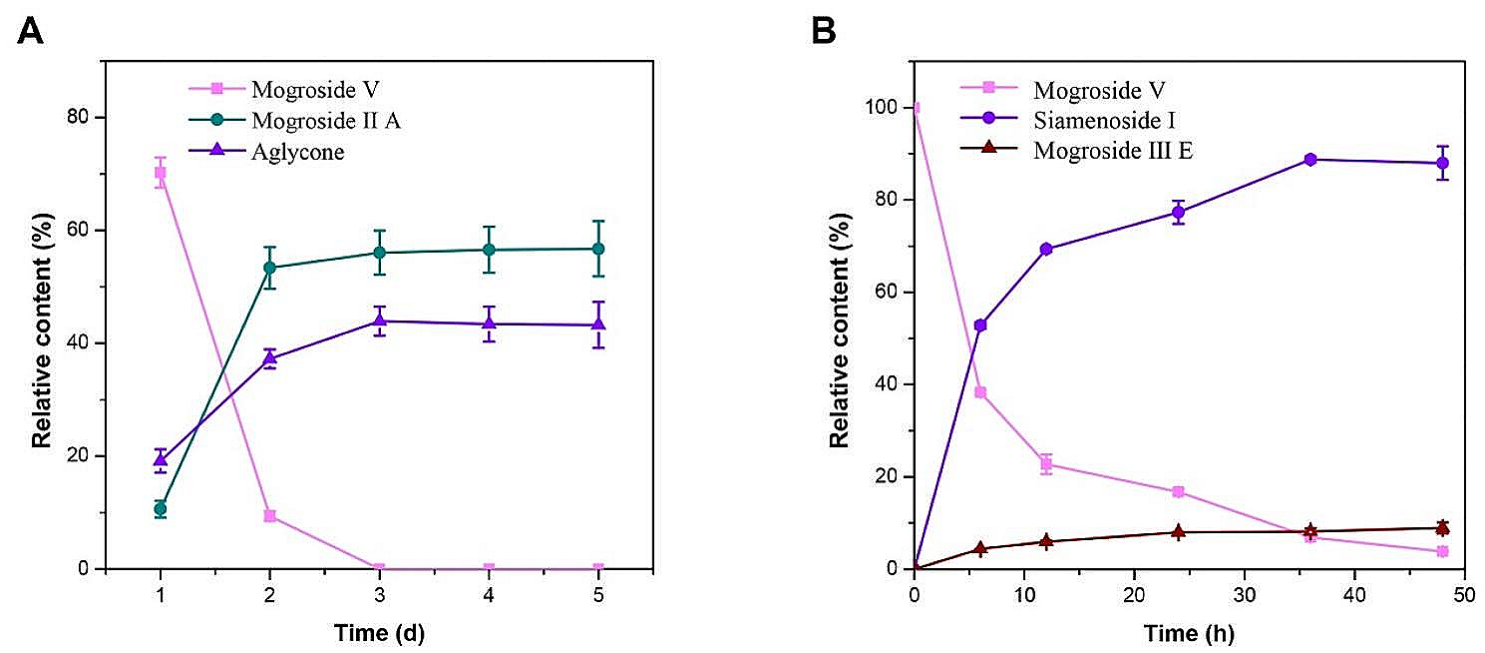
The relative content profiles of mogrosides during bioconversion of **A*** Aspergillus* sp. S125 and **B*** Muyocopron* sp. A5 under optimal fermentation conditions

Subsequently, fermentation of strain A5 and S125 was carried out for rare mogroside production under optimal conditions, respectively (Fig. 5). The HPLC method was further employed to analyze the transformation process of the products. Consistent with the results of the initial screening, *Aspergillus* sp. S125 demonstrated a strong conversion ability with mogroside V, completely transformed in only 3 days, producing 56.07% of mogroside II A and 43.93% of aglycone (S Figs. 2 and 5A). It is worth noting that *Muyocopron* sp. A5 showed different performance in the conversion of mogroside V under optimized fermentation conditions compared to the initial screening. Initially, *Muyocopron* sp. A5 converted mogroside V into various mogrosides, including siamenoside I, mogroside IV E, mogroside III E, and finally mogroside II E (Fig. 1). Surprisingly, after optimization, we found that *Muyocopron* sp. A5 selectively converted mogroside V into siamenoside I during the early stage of fermentation (1–2 days), followed by conversion into mogroside III E after 3 days of fermentation, and ultimately resulting in mogroside II E (S Fig. 3). Therefore, in order to obtain siamenoside I, the fermentation of strain A5 was performed under the optimized conditions for only 48 h. Our results showed that after 36 h of fermentation, the content of siamenoside I accounted for 88.74% of the total mogrosides, and the concentration was 4.88 g/L (Fig. 5B). Wang et al. (2019) screened 5 yeast strains for the conversion of mogroside V and found that siamenoside I accounted for approximately 30–54% of the total glycosides after 7 days of fermentation, along with considerable amounts of Mogroside III E, ranging from 7 to 59%. Compared to other catalytic strains that can convert mogroside V into siamenoside I, plant endophytes *Muyocopron* sp. A5 not only exhibited better selectivity, but also achieved significantly higher efficiency, with 88.74% of siamenoside I obtained in just 36 h. Indeed, plant endophytes *Muyocopron* sp. A5 and *Aspergillus* sp. S125 exhibit higher substrate tolerance and product yield compared to other microbe-mediated mogroside V bioconversions. Our results further suggest the great potential and practical value of endophytic fungi in the biotransformation of mogrosides.

## Conclusions

Selective hydrolysis of the different glucose moieties of mogroside V, the main bioactive compounds of the LHG, is an important and practical strategy for the preparation of other various glycosides. However, the complicated spatial structures of mogroside V pose a challenge for selective hydrolysis of specific glucose moieties. Biocatalytic strategies show significant advantages in preparing rare mogrosides due to their excellent region-selectivity. Therefore, it is critical to screen microorganisms and enzymes that can efficiently and selectively transform mogroside V. In this study, we randomly selected 20 strains from our plant endophytic fungi library and investigated their ability to transform mogroside V using LHG extract (containing 50% of mogroside V) as a substrate. Six strains were found to be capable of transforming mogrosides and producing various products, including siamenoside I, mogroside IV E, mogroside III, mogroside III E, mogroside II A, mogroside II E, mogroside I A, mogroside I E, and aglycone, indicating diverse transformation pathways. Among 6 strains, strain S125 showed efficient transformation ability by completely converting mogroside V in 1–2 days of fermentation, while strain A5 was able to selectively convert mogroside V into a high-intensity natural sweetener siamenoside I. By optimizing the fermentation condition such as substrate addition and nitrogen source, strain S125 was able to completely transform mogroside V in 3 days of fermentation, generating 4.5 g/L of mogroside II A and 3.6 g/L of aglycone. Strain A5 selectively transformed mogroside V into siamenoside I during the early stage of fermentation and was able to convert 93.2% of mogroside V in only 36 h, producing 4.88 g/L of siamenoside I. In addition, morphological and molecular identification of the six plant endophytic fungi strains revealed that multiple strains are potential novel species. This study not only provides diverse candidates for the bioconversion of mogroside which significantly facilitate the development of mogroside industry, but also demonstrates that endophytic fungi are a great potential biocatalytic resource for natural products.

### Electronic supplementary material

Below is the link to the electronic supplementary material.

Supplementary Material 1

Supplementary Material 2

## Data Availability

Not applicable.
