# Supplementary material for: Plant endophytic fungi exhibit diverse biotransformation pathways of mogrosides and show great potential application in siamenoside I production
Source: Bioresour Bioprocess. 2024 Apr 23;11(1):42. doi: 10.1186/s40643-024-00754-8 (PMC11039582; doi:10.1186/s40643-024-00754-8)
Supplement: Supplementary file 1 — Supplementary Material 1 [file 40643_2024_754_MOESM1_ESM.docx]

**
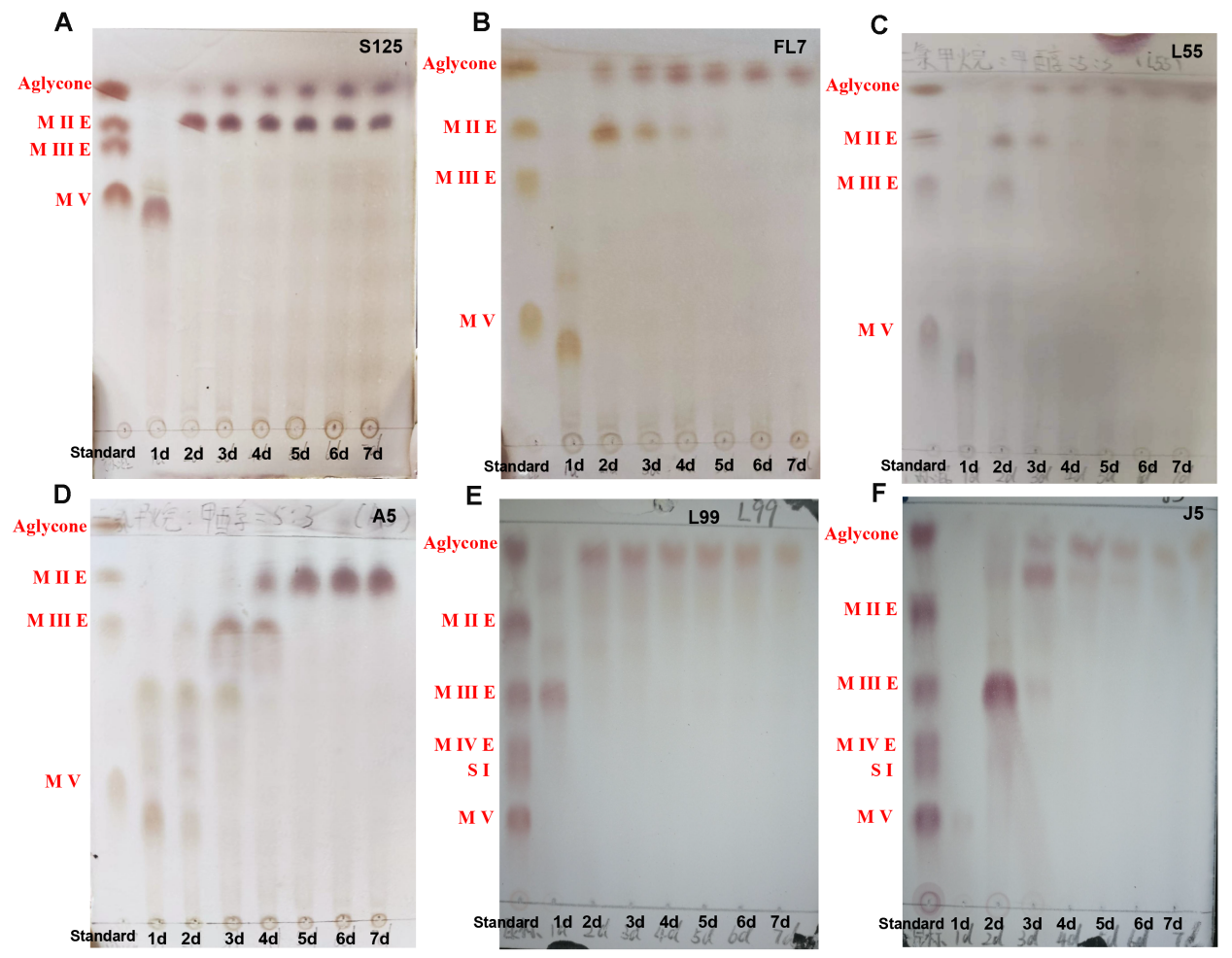
S Fig. 1** The thin layer chromatographic plate (TLC) analysis was carried out for the analyzing products which produced by the bioconversion of given 6 fungal endophytes. The fungal mycelium of strain S125, FL7, L55, A5, L99 and J5 were inoculated with 2% (v/w) into inorganic medium containing 5% (w/v) LHG extract as the only carbon source, and aerobic fermentation was carried out at 160 rpm in a shaker at 28 ℃ for 7 days, respectively. After fermentation, the broth was sampled per 12 h, and the supernatant of strain S125 (A), FL7 (B), L55 (C), A5 (D), L99 (E) and J5 (F) was collected by centrifugation at 4 ℃, 7, 000 rpm, and directly analyzed by the TLC method.


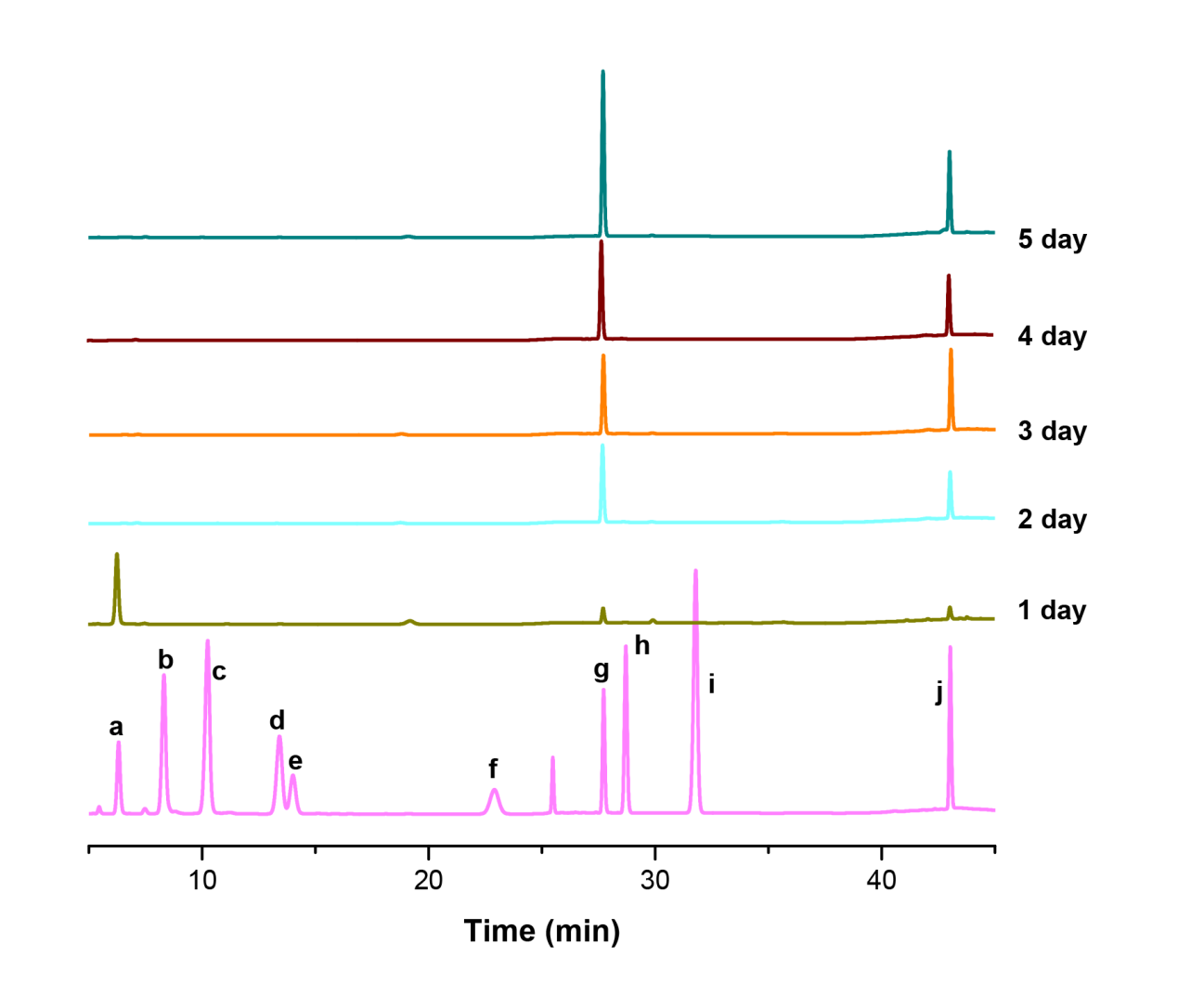


**S Fig. 2** The HPLC method was performed for analyzing conversing products of *Aspergillus* sp. S125. The standards as following: a. mogroside Ⅴ; b. siamenoside Ⅰ; c. mogroside Ⅳ E; d. mogroside Ⅲ; e. mogroside Ⅲ E; f. mogroside Ⅱ E; g. mogroside Ⅱ A; h. mogroside Ⅰ E; i. mogroside Ⅰ A; j. glycone.

**
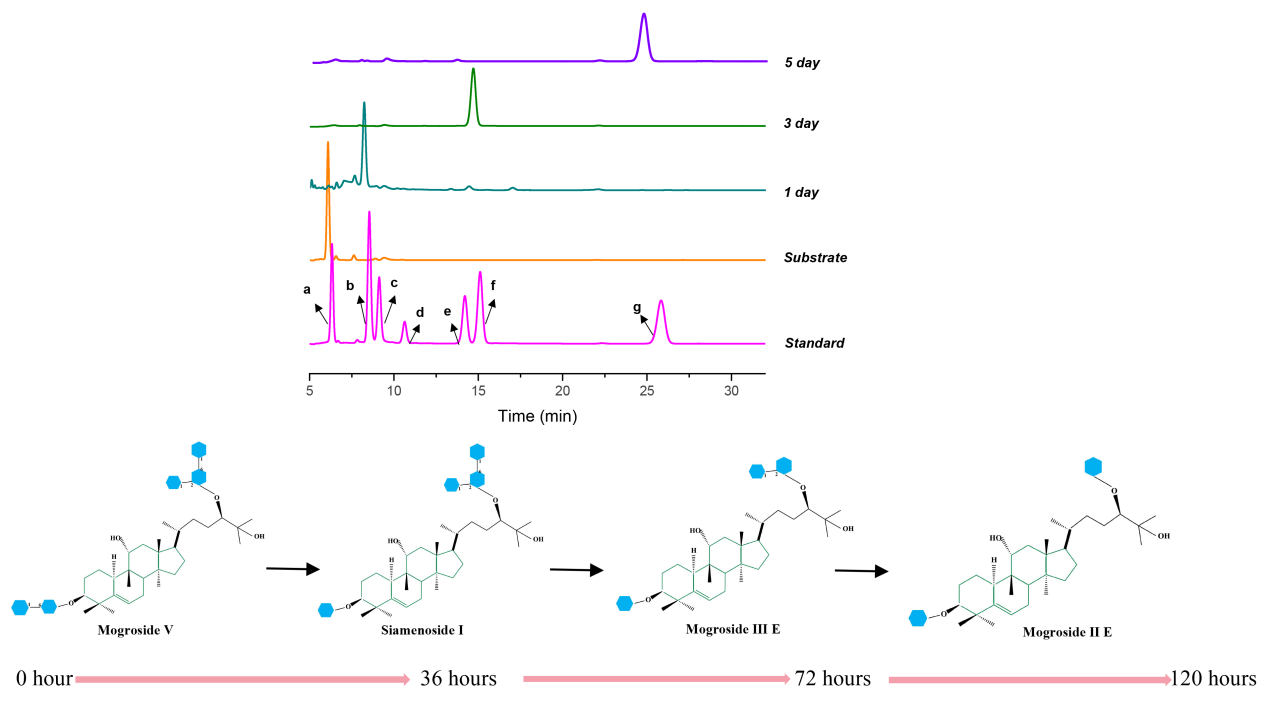
S Fig. 3** The HPLC method was performed for analyzing conversing products of *Muyocopron* sp. A5. The standards as following: a. mogroside Ⅴ; b. siamenoside Ⅰ; c. mogroside Ⅳ A; d. mogroside Ⅳ E; e. mogroside Ⅲ; f. mogroside Ⅲ E; g. mogroside Ⅱ E.
