# Supplementary figures and images for: Plant endophytic fungi exhibit diverse biotransformation pathways of mogrosides and show great potential application in siamenoside I production
Source: Bioresour Bioprocess. 2024 Apr 23;11(1):42. doi: 10.1186/s40643-024-00754-8 (PMC11039582; doi:10.1186/s40643-024-00754-8)

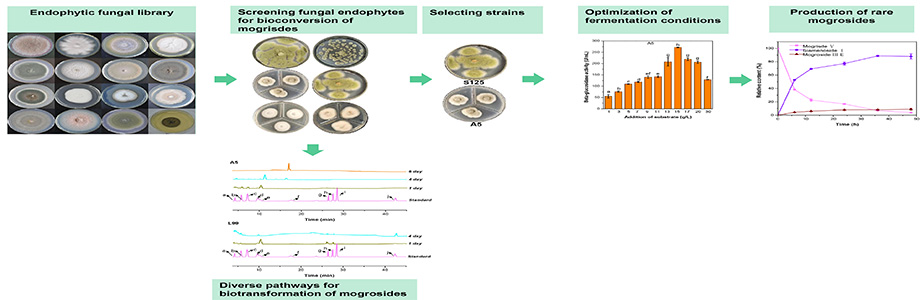

Supplement: Supplementary file 2 — Supplementary Material 2 [file 40643_2024_754_MOESM2_ESM.jpg]
